# Hospitals and the Law

**Published:** 1911-04-15

**Authors:** 


### HOSPITALS AND THE LAW.

### THE RATING OF HOSPITALS.

A case relating to the rating of the Royal Bath Hospital and Rawson Convalescent Home, Harrogate, which was heard in the Divisional Court on March 31, raised a point of general interest to hospitals.

The appeal was by the Knaresborough Union, which asked the Court to set aside the decision of the Justices of the West Riding Quarter Sessions, who had reduced the assessment of the buildings and land made in April 1910 from £727 gross estimated rental and £581 net rateable value, to £50 gross rental and £40 net rateable value.

The Justices stated a special case for the opinion of the High Court, and the document contained many interesting facts as to the establishment of the hospital and home and the objects of the institution. It appeared that the hospital was founded in 1824, the site having been given by Lord Harewood, but it was not until 1888 that the present buildings were erected on the original site and additional land given by his lordship. The cost of the building amounted to £36,492, exclusive of the land. This amount was raised entirely by bequests and subscriptions. The property is vested in trustees, and the hospital was founded for the relief of poor persons who are proper objects of charity, the benefits being conferred upon such people free of expense. The convalescent home was founded in like manner for the use of persons who could not otherwise avail themselves of the air and water of Harrogate, applications being accepted only from persons in indigent circumstances and of good character. The institutions are supported by subscriptions, forming an endowment fund. The sum received from invested funds amounted to £827 per annum, the home receiving about £200 from fees and £1,745 from subscriptions. People from all parts of the British Isles are admitted.

It was contended on behalf of the appellant Union at the Quarter Sessions that the buildings and land were liable to be rated; that the respondents were in beneficial occupation; that when the owners were in occupation the percentage of the structural value was evidence of the rent which the occupants as tenants might reasonably be expected to pay, and that the appellants, by taking 2 per cent. of the structural value of the place, adopted a fair and reasonable method of arriving at the value of the hereditaments. Therefore, it was contended that the true amount of the structural value of the buildings was £28,350 and of the land £8,046, making an estimated gross rental of £727. The Court of Quarter Sessions, however, were of opinion that the hospital and home should be rated at £50 gross and £40 net rateable value.

About a dozen other hospital cases in the Union depended on this, and it was for this reason that it was brought before the High Court; but as the Lord Chief Justice held that no point of law had arisen before the Court of Quarter Sessions, he could not interfere. Indeed, it was pointed out that the chairman of Sessions stated that the Court would not commit itself to any statement, but simply named the sum at which the buildings should be assessed.

As this case was decided on its own peculiar facts it cannot be stretched very far as an authority. Nevertheless, it is a recognition of the principle that for the purposes of rating, a charitable institution, such as a hospital, the rate is not to be founded on a mere percentage of the value or cost of the buildings.

